# Patient-centered inpatient psychiatry is associated with outcomes, ownership, and national quality measures

**DOI:** 10.1093/haschl/qxad017

**Published:** 2023-06-20

**Authors:** Morgan C Shields, Mara A G Hollander, Alisa B Busch, Zohra Kantawala, Meredith B Rosenthal

**Affiliations:** Brown School, Washington University in St. Louis, St. Louis, MO 63130, United States; Department of Public Health Sciences, University of North Carolina at Charlotte, Charlotte, NC 28223, United States; Department of Health Care Policy, Harvard University, Harvard Medical School, Boston, MA 02115, United States; McLean Hospital, Belmont, MA 02478, United States; Brown School, Washington University in St. Louis, St. Louis, MO 63130, United States; Department of Health Policy and Management, Harvard University, Harvard T. H. Chan School of Public Health, Boston, MA 02115, United States

**Keywords:** quality, mental health care, policy, public reporting, patient experience

## Abstract

Following discharge from inpatient psychiatry, patients experience elevated suicide risk, unplanned readmission, and lack of outpatient follow-up visits. These negative outcomes might relate to patient-centered care (PCC) experiences while hospitalized. We surveyed 739 former patients of inpatient psychiatric settings to understand the relationship between PCC and changes in patients’ trust, willingness to engage in care, and self-reported 30-day follow-up visits. We also linked PCC measures to facility-level quality measures in the Inpatient Psychiatric Facility Quality Reporting program. Relative to patients discharged from facilities in the top quartile of PCC, those discharged from facilities in the bottom quartile were more likely to experience reduced trust (predicted probability [PP] = 0.77 vs 0.46; *P* < .001), reduced willingness to go to the hospital voluntarily (PP = 0.99 vs 0.01; *P* < .001), and a lower likelihood of a 30-day follow-up (PP = 0.71 vs 0.92; *P* < .001). PCC was lower among patients discharged from for-profits, was positively associated with facility-level quality measures of 7- and 30-day follow-up and medication continuation, and was inversely associated with restraint use. Findings underscore the need to introduce systematic measurement and improvement of PCC in this setting.

## Introduction

Research on the quality of inpatient psychiatric care has been lacking, despite documented concerns that care in these settings can be unsafe and misaligned with patients’ needs and preferences.^1–6^ The causes of safety events can range from medication error to the use of restraint and seclusion,^2,3,7–10^ and are likely experienced disproportionally by minoritized patients.^11^ Some people who have experienced psychiatric hospitalization have reported positive experiences, while others have reported disrespectful and dehumanizing care,^12^ and news reports have illuminated an array of issues related to substandard care.^7^

People discharged after inpatient psychiatry experience a suicide rate within 7 and 30 days postdischarge estimated to be approximately 300 and 200 times the general suicide rate, respectively.^13^ These high suicide rates following discharge from inpatient psychiatry have led some researchers to hypothesize that psychiatric hospitalization might have an iatrogenic effect among some patients.^1,14^ While connecting individuals to follow-up care within the first month of discharge can support access to effective treatment and reduce the risk of homelessness, readmission, and suicide,^15–20^ only about half of discharged patients have a follow-up visit within 30 days. These increased risks for adverse outcomes and lack of engagement with postdischarge care might relate to the degree to which patients perceive their care as patient-centered^6,21,22^—that is, care that is respectful, responsive to patients’ needs and preferences, transparent, and coordinated.

### Patient-centered care

Patient-centered care (PCC) is 1 of the 6 domains of quality outlined by the Institute of Medicine, representing an endpoint in its own right.^23^ While payers, policymakers, and researchers have focused on supporting and understanding the implementation of PCC principles and behaviors in general health care settings, inpatient psychiatry has been left on the sidelines of these initiatives.^2^ For example, psychiatric patients are the only population excluded from the Hospital Consumer Assessment of Healthcare Providers and Systems (HCAHPS), a national survey of hospitalized patients’ experience of care. The current HCAHPS measure used in general hospital care may not be appropriate to capture the distinct care configurations and workflows in inpatient psychiatry. However, the exclusion of psychiatric patients from measurement and reporting of patient experience forecloses opportunities to conduct systems-level research to understand how patient-centered inpatient psychiatric care varies within and across hospitals, to estimate the association of PCC with outcomes, and to spark improvement along this critical dimension of quality.

### The unique context of inpatient psychiatry

Inpatient psychiatric patients are hospitalized primarily for concerns related to psychological and emotional distress, rather than physical ailments, and the treatment environment is congregate by design. Therefore, both treatment and unit management in these settings rely on skills that are interpersonal in nature. Indeed, evidence-based models for preventing trauma, violence, restraint, and seclusion in inpatient psychiatric care settings are founded on patient-centered, trauma-informed care principles.^24^

Patients of inpatient psychiatry report experiences of humiliation and a loss of freedom and agency,^4,5,25^ which might occur against the backdrop of related social crises outside the hospital's walls (eg, the recent loss of employment/income, fractured relationships, loss of housing, pending criminal charges). Prior trauma is also highly prevalent among this patient population, with the treatment environment posing risks for re-traumatization.^5^ For example, considerable power imbalances between patients and providers are enabled by market failures, including patients’ lack of information about the quality of care they will receive before admission and limits in their ability to choose whether and where to be hospitalized. These market failures mean that inpatient psychiatric facilities are not rewarded or punished by the “consumers” of their services (ie, patients) based on the quality of care they provide; patients do not produce the same type of demand response that consumers of shoes do, for example.^26^ Further, engagement from loved ones during visitation can provide an alternative source of advocacy for patients to ensure quality (through consistent, external observation); however, on inpatient psychiatry units (compared with other medical units), loved ones often have greater restrictions in their ability to “be at the bedside” of patients, with requirements such as coming during prescribed hours and meeting with patients in a separate space from the common areas. Patients may face significant barriers to speaking up due to diminished credibility, fear of retaliation, stigma, and limited social capital.^2,7^

### Federal quality reporting program for inpatient psychiatry

The Centers for Medicare and Medicaid Services (CMS) implemented the Inpatient Psychiatric Facility Quality Reporting (IPFQR) program at the end of 2012 as a first step towards accountability of inpatient psychiatric care organizations. While the measures in the IPFQR program have fluctuated over the years, they currently include restraint and seclusion, care processes (eg, screening for tobacco use), and claims-based measures of 7- and 30-day follow-up, 30-day all-cause readmission, and postdischarge medication continuation. There are currently no patient experience measures included in the IPFQR. It is unknown to what extent the current IPFQR measures are associated with PCC.

### The role of tax status

An important part of the current policy context for inpatient psychiatry is the rise in market share controlled by for-profit systems and chains.^27^

The implications of for-profit ownership on care quality are unclear. Economic theory suggests that for-profit entities prioritize profits and may leverage market failures to do so,^26^ and robust empirical evidence among other health care settings demonstrates lower quality among for-profit settings than nonprofits.^28^ One of the largest suppliers of psychiatric beds in the country (a for-profit company) has faced numerous lawsuits and media coverage regarding issues of substandard and unsafe care—with reports suggesting that the company prioritized profits at the expense of quality (eg, employing unqualified and unsupervised staff).^2,7^

Empirical analyses describing variation in IPFQR measure performance across ownership categories have found that for-profits perform on par or better compared with nonprofit and government-owned facilities.^29,30^ It is unclear, however, whether performance on the IPFQR measures is associated with better patient experiences of care, and how these experiences might differ across ownership types.

### Current study

In the current study, we examined the association of psychiatric patients’ experiences of PCC with self-reported changes in trust, willingness to engage in postdischarge care, overall impact of care, and 30-day follow-up visits. We hypothesized that higher levels of PCC would be positively associated with patient-reported outcomes. In addition, we hypothesized that for-profits would have the lowest reports of PCC.

Given the saliency of this issue to evolving accountability programs, we also examined the associations between patients’ reported experiences of PCC and their respective hospital's performance on IPFQR measures. We hypothesized that IPFQR measures that are self-reported by facilities would be less likely to be positively associated with PCC, as these measures had the greatest opportunity to be gamed, to have been reported with error, and could have been performed on without meaningful improvement in the care environment. In contrast, we hypothesized that claims-based measures (those calculated by CMS using their administrative data rather than self-reported by facilities) would be positively associated with reports of PCC.

To our knowledge, this is the first empirical analysis to examine the relationship between patients’ reports of patient-centered inpatient psychiatric care and changes in their trust, engagement with postdischarge care, and ownership, as well as to study the relationship between reports of PCC and facility-level quality measures used in current accountability programs.

## Data and methods

### Study design and data sources

Utilizing a retrospective cohort design, we administered an online survey in early 2021 to adults who had experienced an inpatient psychiatric admission. We linked responses to this survey to facility-level information on ownership and facility type using Medicare's Provider of Services (POS) file and online searches. We also linked survey responses to the IPFQR quality measures, which are publicly reported at the facility level.

Participants were invited to participate in the survey through diverse outlets such as Twitter, Facebook, Instagram, and Reddit. We chose this recruitment strategy given a lack of a recruitment frame outside of single-site locations, and because we were primarily interested in recruiting representation across the spectrum of care experiences. Wide variation in experiences best allowed us to test the associations between PCC and outcomes.

Participants were screened for eligibility based on the following inclusion criteria: (1) having had at least 1 psychiatric hospitalization between 2016 and 2021, (2) being aged 18 or older at the time of their most recent psychiatric hospitalization, and (3) their most recent psychiatric hospitalization took place in the United States. Through the survey, participants were asked a series of demographic questions and were then prompted to respond to several questionnaires oriented around their most recent psychiatric hospitalization. For respondents who provided enough information about the location of their hospitalization (name, city, and state of the facility), we linked these responses to facility-level characteristics and quality performance. The survey took up to 20 minutes to complete. At the end of the survey, participants could enter a lottery to win a $25 gift card. This study was approved by The Institutional Review Board of the University of Pennsylvania (#844878).

### Survey measures

#### Measures of patient characteristics

Measures of clinical and demographic patient characteristics included the following categorical variables: age (18–24, 25–34, 35–44, >44 years), gender (female, male, nonbinary/third-gender/other), race (non-Hispanic/Latinx White, Native, Hawaiian/Pacific Islander, Black, Asian, multiple races, “other”), Hispanic/Latinx (yes/no), education (high school degree or less, some college/associate’s degree/trade school, 4-year degree, advanced degree), annual income (<$25 000, $25 000–$49 999, $50 000–$99 999, >$99 999), had insurance at time of hospitalization (yes/no), number of psychiatric hospitalizations (1, 2, 3, 4–6, 7–9, >9), hospitalized for suicidal ideation or behavior (yes/no), involuntary/did not want to be hospitalized (yes/no), and hospitalized during the COVID-19 era (yes/no). Participants were also asked to respond to 5-point Likert items that assessed baseline expectations in quality of care (the degree to which they expected to receive safe and high-quality care at the time of admission).

#### Measure of patient-centered care (primary predictor)

Patient-centered care was measured using a valid and reliable instrument, the Combined Assessment of Psychiatric Environments (CAPE).^31^ The CAPE consists of 24 questions on a 4-point Likert scale (0 = never; 1 = sometimes; 2 = usually; 3 = always) and comprises 2 domains (staff competence and treatment efficacy). We created a summary score of PCC for each respondent from the CAPE responses. Scores could range from 0 to 72. From the summary score, we assigned scores into quartiles.

#### Measures of patient-level outcomes

Participants were asked to respond to 5-point Likert items, which were then converted into binary indicators, that assessed the extent to which the hospitalization reduced or increased their trust in mental health providers, the extent to which the hospitalization reduced or increased their willingness to disclose distressing thoughts to outpatient providers, and the extent to which the hospitalization reduced or increased their willingness to voluntarily go to the hospital in the future when in psychological distress. Participants also provided a global assessment of the degree to which the hospitalization had a negative or positive impact on them (positive, negative, mixed, neutral), and if they received follow-up care within 30 days of discharge.

#### Measures of facility-level characteristics

Facility characteristics included ownership (for-profit, nonprofit, government) and facility type (unit of a general hospital vs freestanding psychiatric facility). These measures came from the POS file, a dataset previously used in research on inpatient psychiatric facilities.^32^ We used CMS’s IPFQR quality measures posted publicly in 2021, reflecting 2019 performance. See the program's specification manual for more details.^33^

### Analysis

To identify the relationship between PCC and outcomes, we fit 9 mixed-effects logistic regression models, including a random intercept for the hospital to account for the known clustering of respondents associated with the same hospital. For responses that we could not link to specific hospitals, we treated these as distinct clusters. Models controlled for participants’ baseline expectations in the quality of hospital care at the time of admission, voluntary status, suicidality, prior psychiatric admissions, demographic characteristics, and year of admission. We then produced predicted probabilities of each outcome across quartiles of PCC, with covariates held at their observed values. Given prior research demonstrating differences in patients’ experiences of quality based on their perceptions of coercion,^34,35^ we tested for interactions between voluntary status and PCC quartiles.

Among respondents who provided enough information about the location of their hospitalization and could be linked to facility-level information, we fit another series of mixed-effects linear regression models examining the relationship between PCC and the respective hospital's ownership, type, and performance on the IPFQR quality measures. For all of these models, we controlled for patient characteristics. We report mean predicted values of PCC across ownership and facility type and linear regression coefficients associated with each quality measure and PCC.

### Limitations

Results from this study should be interpreted in light of several limitations. First, we utilized a convenience sample of former patients recruited online. However, the primary aim of the study was to understand relationships rather than to estimate national rates of PCC. Thus, variation in PCC, the predictor of interest, was of greater importance; we observed wide variation in PCC within the data. When compared with national data on inpatient psychiatric patients in general hospitals, our sample reflects a similar distribution of the top 3 diagnoses (depression, bipolar, and schizophrenia), although it skews younger.^36^ Second, our measure of trust was not validated, suggesting an area needing further measure development, and the retrospective design introduces the possibility of recall bias. Third, the identified associations between PCC and facility-level characteristics and quality measures should be interpreted with caution given that PCC and the facility-level measures were assessed at different levels (patient vs facility) and our data prevented us from distinguishing within-facility versus between-facility variation. Nevertheless, research has consistently suggested that differences in quality of care are largely driven by institutional factors (eg, hospital culture, staffing, financing); there is no reason to expect this phenomenon to differ in the inpatient psychiatry context.

## Results

After excluding respondents who did not meet inclusion criteria, did not complete the survey, or appeared to be bots, there were 739 survey responses, 441 (59.7%) of which were linked to ownership and facility type and 333 of which (45.1%) were linked to IPFQR quality measures. Sample characteristics are displayed in Table 1. The majority of respondents were younger than 35 years (76.3%). Nearly half (47.5%) were female. Approximately two-thirds (63.2%) were non-Hispanic White; another fifth were Hispanic/Latinx (20.2%). One-quarter (27.7%) had a 4-year college education and one-third (33.2%) made less than $25 000 per year.

**Table 1.** Sample characteristics (N = 739).

	n	%
Age (y)		
18–24	281	38.02
25–34	283	38.29
35–44	107	14.48
>44	68	9.20
Gender		
Female	351	47.50
Male	334	45.20
Nonbinary, third-gender, or other	54	7.31
Race/ethnicity		
Non-Hispanic White	467	63.19
Non-Hispanic Native American	30	4.06
Non-Hispanic Hawaiian/Pacific Islander	15	2.03
Non-Hispanic Black	23	3.11
Non-Hispanic Asian	22	2.98
Non-Hispanic “other”	7	0.95
Non-Hispanic multiple races	26	3.52
Hispanic/Latinx	149	20.16
Education		
High school degree or less	125	16.91
Some college/associate’s degree/trade school	341	46.14
Four-year college degree	205	27.74
Advanced degree (master's, MD, JD, PhD	68	9.2
Income		
<$25 000	245	33.15
$25 000-$49 999	250	33.83
$50 000-$99 999	170	23.00
>$99 999	74	10.01
Had insurance	670	90.66
Number of psychiatric hospitalizations		
1	252	34.10
2	167	22.60
3	127	17.19
4–6	111	15.02
7–9	34	4.59
>9	48	6.54
Hospitalized for suicidal ideation or behavior	505	68.34
Involuntary/did not want to be hospitalized	325	43.98
Hospitalized during the COVID-19 era	160	21.65

Source: Data are from an online survey administered to former inpatient psychiatry patients in 2021.

Most (90.7%) respondents reported having insurance at the time of their hospitalization. About one-third of respondents reported having experienced only 1 psychiatric hospitalization in their lifetime. Two-thirds (68.3%) were hospitalized for suicidal ideation or behavior, and nearly half (44.0%) were hospitalized involuntarily. Respondents were more likely to provide their location of hospitalization—enabling us to link their survey responses to the IPFQR quality measures—if they were female, non-Hispanic White, more highly educated, and higher income respondents. There was significant variation in PCC (see Supplemental Material Figure S1).^37^

**Figure 1.**
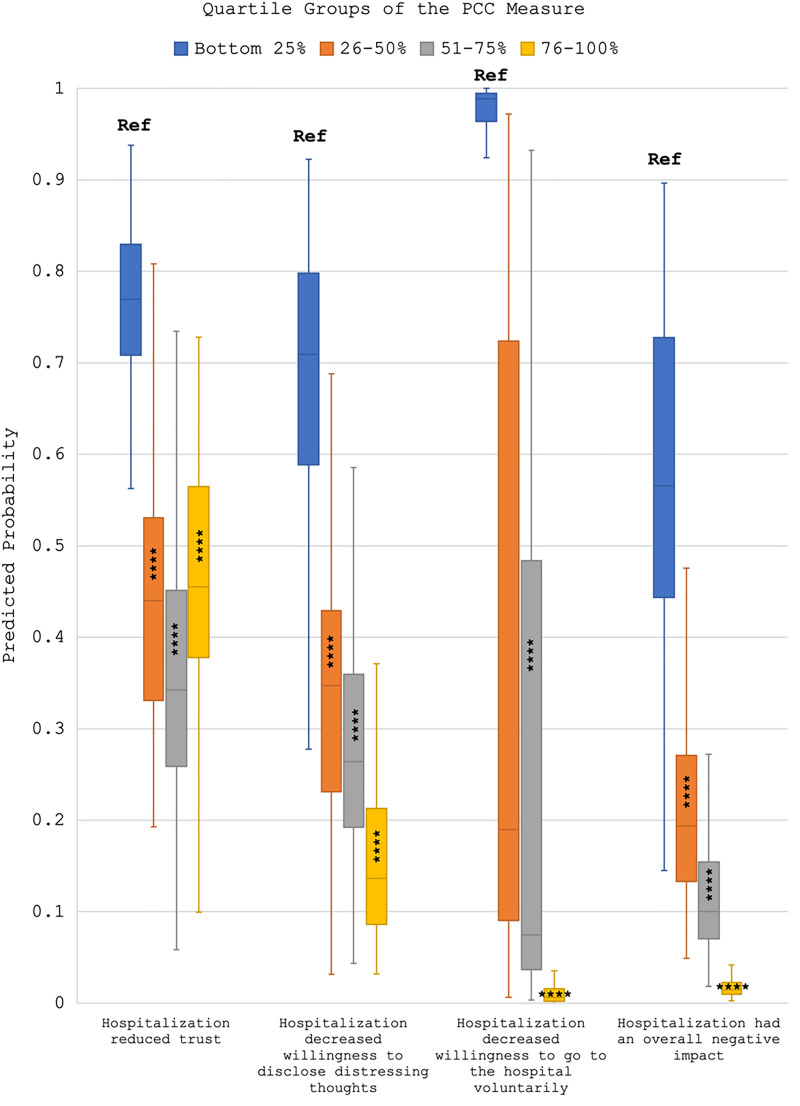
Box plots of predicted probabilities from adjusted regression models of PCC on negative patient-reported outcomes. Source: Data are from an online survey administered to former inpatient psychiatry patients in 2021. Predicted probabilities come from 4 regression models (n = 739). Full models are provided in the Supplemental Material Table S2. Box plots represent the interquartile range in the colored box, with the median at the point of the line; the tails indicate the maximum and minimum probabilities. ****P < .001. Abbreviations: PCC, patient-centered care; Ref, reference group.

**Figure 2.**
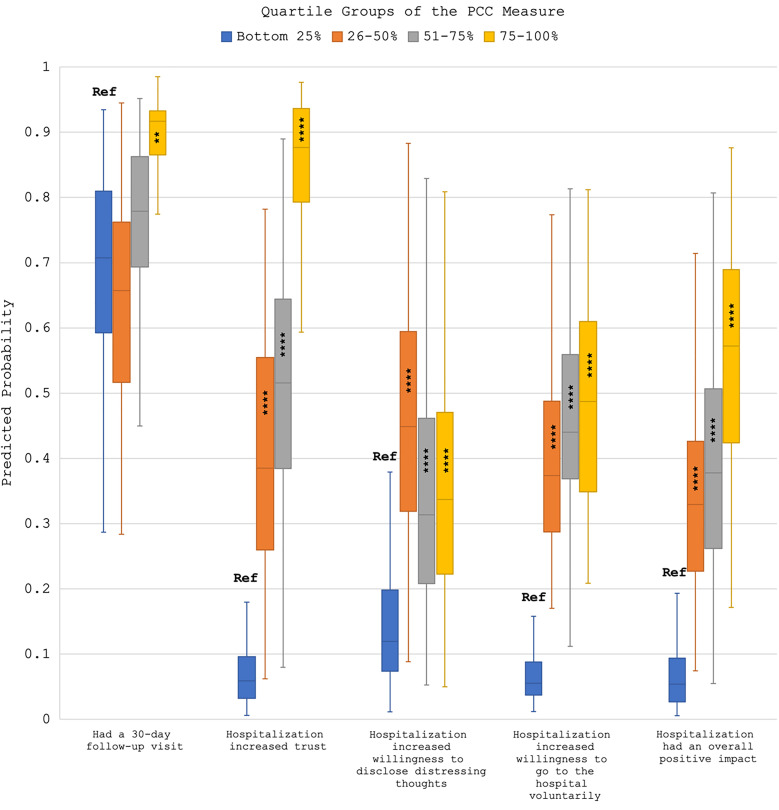
Box plots of predicted probabilities from adjusted regression models of PCC on positive patient-reported outcomes. Source: Data are from an online survey administered to former inpatient psychiatry patients in 2021. Predicted probabilities come from 5 regression models (n = 739). Full models are provided in the Supplemental Material Table S3. **P < .05; ****P < .001. Box plots represent the interquartile range in the colored box, with the median at the point of the line; the tails indicate the maximum and minimum probabilities. Abbreviations: PCC, patient-centered care; Ref, reference group.

In Figures 1 and 2, we present differences in predicted probabilities of specific outcomes occurring across quartiles of PCC using box plots. We found a relationship between quartiles of PCC and outcomes in the expected direction. For example, among those reporting PCC in the bottom quartile, there was a 98.8% median probability that the hospitalization decreased their willingness to voluntarily go to the hospital in the future for psychological distress, but this probability dropped to just 0.6% for those in the top quartile of PCC. Likewise, those in the bottom quartile of PCC had a 70.7% probability of reporting a 30-day follow-up visit, compared to a 91.7% probability among those in the top quartile of PCC. See Supplemental Material Tables S2 and S3 for full models, and Supplemental Material Tables S4 and S5 for tables of predicted probability means and their respective 95% confidence intervals.^37^ We did not find evidence for differences in relationships by voluntary status.

Table 2 reports results from regression models demonstrating the relationship between PCC and hospital ownership, facility type, and performance on the IPFQR measures. PCC was lower among respondents who received care at a for-profit (predicted mean = 31.67) compared with a nonprofit (predicted mean = 40.41; *P* < 0.001) facility. PCC was not associated with being admitted to a unit of a general hospital or freestanding psychiatric facility. PCC was inversely associated with restraint (β = 0.019, *P* < .10) and was positively associated with facility-level quality measures: screening for metabolic disorders (β = 0.28, *P* < .01), offering tobacco treatment at discharge (β = 0.22, *P* < .1), receipt of transition record with required elements (β = 0.26, *P* < .05), timely transition of the transition record (β = 0.27, *P* < .05), and influenza immunization (β = 0.12, *P* < .1). Among the claims-based quality measures, PCC was associated with 7-day (β = 0.19, *P* < .001) and 30-day (β = 0.19, *P* < .001) follow-up visits, as well as patients’ continuation of medication following discharge (β = 0.06, *P* < .05).

**Table 2.** Associations between patient-reported PCC and facility-level characteristics and performance on CMS’s IPFQR quality measures.

	Predicted PCC Mean	95% CI		*P*
Facility characteristics associated with PCC				
Ownership and facility type				
For-profit	31.67	29.92	33.43	Ref
Nonprofit	40.41	39.24	41.58	****
Government	37.74	35.40	40.09	
Facility type				
Freestanding psychiatric hospital	36.11	34.71	37.51	Ref
Unit in a general hospital	38.84	37.53	40.14	
	**Linear regression coefficient**			
IPFQR facility-level quality measures associated with PCC				
* *Chart-abstracted process measures				
Restraint use	−0.019	−0.038	0.000	*
Seclusion use	0.014	−0.005	0.034	
Screening for metabolic disorders	0.283	0.112	0.454	***
Tobacco use treatment provided or offered	0.013	−0.136	0.162	
Tobacco use treatment while hospitalized	0.120	−0.065	0.305	
Tobacco use treatment provided or offered at discharge	0.218	0.005	0.431	*
Tobacco use treatment at discharge	−0.060	−0.242	0.121	
Transition record with specified elements received by discharged patients	0.259	0.047	0.470	**
Timely transmission of transition record	0.269	0.059	0.479	**
Influenza immunization	0.124	−0.009	0.257	*
Claims-based outcomes measures				
7-Day follow-up visit for mental illness	0.190	0.109	0.270	****
30-Day follow-up visit for mental illness	0.185	0.099	0.271	****
30-Day all-cause unplanned readmission following psychiatric hospitalization in an inpatient psychiatric facility	−0.013	−0.032	0.005	
Medication continuation following inpatient psychiatric discharge	0.063	0.013	0.112	**

Source: Patient-level data on PCC are from an online survey administered to former inpatient psychiatry patients in 2021. Facility-level quality measures come from the CMS’s 2021 Inpatient Psychiatric Facility Quality Reporting Program. Data on facility type and ownership are from Medicare's Provider of Services file and online searches. Data on ownership were available for n = 454. Data on facility type were available for n = 437. Linkages to facility-level quality measures were available for n = 333. All models controlled for patient characteristics. *P < .1 **P < .05; ***P < .01; ****P < .001.

Abbreviations: CMS, Centers for Medicare and Medicaid Services; IPFQR, Inpatient Psychiatric Facility Quality Reporting; PCC, patient-centered care; Ref, reference group.

## Discussion

Consistent with our hypotheses, psychiatric patients’ reports of PCC were associated with changes in trust and postdischarge engagement with care. PCC was also associated with many facility-level quality measures, such as 7- and 30-day follow-up rates and patients’ continuation of medications postdischarge. PCC was higher among patients who received care at nonprofit facilities than at for-profit facilities. These are the first known findings demonstrating a relationship between PCC in inpatient psychiatry and patient-reported postdischarge outcomes. To our knowledge, this is also the first study to examine and demonstrate a relationship between PCC and ownership type, as well as PCC and performance on national quality measures.

There has been limited research to examine PCC's relationship with outcomes from inpatient psychiatry. One 2011 study linked patients’ perceptions of quality to satisfaction and trust but did not measure willingness to engage in postdischarge care and follow-up visits.^38^ Moreover, most interventions to improve postdischarge follow-up visits have focused almost exclusively on discharge planning,^39,40^ rather than care experiences during a patient's tenure of hospitalization. While there are a range of external factors that might influence postdischarge utilization, including insurance coverage, transportation, and availability of outpatient services, our findings suggest that improving PCC may be another mechanism through which to improve engagement with postdischarge care.

An important finding from this study was that the association of PCC with outcomes was independent of a variety of patient characteristics. Of particular note, PCC was associated with outcomes regardless of patients’ voluntary status and expectations of care before admission. This finding is significant given that a concern people have voiced about the use of patient experience measures in this setting is that patients’ evaluation of their care might not be independent of their agreement to care or their overall outlook/expectations of care. Our findings bolster the credibility of patient experience measures as quality indicators in this patient population and suggest that improving PCC can benefit both voluntary and involuntary inpatients.

Patient-reported PCC was also associated with facility-level quality measures, and particularly those measures that were claims-based and capture postdischarge utilization with care (ie, 7- and 30-day follow-up visits, medication continuation). These associations not only mirror the associations found among patient-level outcomes (ie, trust, willingness to engage in care, and 30-day follow-up visits), but this concordance suggests that both the PCC measures and many of the IPFQR measures—especially the claims-based measures—are valid signals of care quality. It is unclear to what extent these relationships are driven by an underlying construct of quality, where “high quality” facilities provide higher levels of PCC while also performing well on other relevant domains of care quality and postdischarge planning, or if it is the case that PCC is a meaningful mechanism through which hospitals influence postdischarge outcomes. Future research is needed to identify the mediating role of PCC on outcomes.

Restraint use was inversely associated with PCC, which is unsurprising given that PCC environments are critical to evidence-based models for preventing the use of restraint and seclusion. However, seclusion was among the few IPFQR measures that was not associated with PCC. Prior work has documented errors with these restraint and seclusion measures.^30^ As such, it is unclear to what extent a lack of association between PCC and seclusion use reflects measurement error or true independence.

Another notable finding was that patients who received care at for-profit facilities reported lower rates of PCC compared with those receiving care at nonprofits. Existing research examining differences in the quality of inpatient psychiatry across ownership types has been limited to the use of process-based measures, finding either no difference between for-profits and nonprofits or superior performance among for-profits.^29^ It could be that certain process-based measures do not provide a valid signal of quality, or that those aspects of quality are not associated with the specific domain of PCC. To the extent that process-based measures can be gamed or performed on without meaningful investment into quality, some process-based measures might create a false reputational image and generate misguided trust and assurance across stakeholders (consumers, families, payers, regulators, policymakers, and advocates). According to economic theory, we would expect for-profits to be more likely than nonprofits to identify ways to perform on quality measures without meaningfully improving care experiences for patients.^26^ Given the rise in for-profit ownership of inpatient psychiatric facilities,^27^ it is imperative that researchers and policymakers identify robust ways to hold inpatient psychiatric care providers accountable.

While we have demonstrated in this study that PCC is associated with quality indicators of interest to stakeholders, it is important to interpret these results with an appreciation for PCC as an important endpoint, regardless of the mediating role it might play in other quality indicators. Empirical findings from this study should underscore the worthwhileness of centering PCC in accountability and quality-improvement initiatives for inpatient psychiatry. PCC is most likely a more meaningful signal for care quality in the setting of inpatient psychiatry,^6^ while also being harder to game, than many measures in the current IPFQR program. This is particularly true when considering that the presenting clinical concern of patients in these settings is emotional and psychological in nature, and appropriate clinical care requires strong relational skills. Further, the power imbalance between provider and patient plays an even larger role in this setting.^2^

One step towards supporting future research in this area, while also moving policy towards centering PCC in this setting, is to expand the sampling of patients for the HCAHPS survey to include psychiatric patients. This would allow us to estimate how PCC differs between psychiatric and other medical units (at least in terms of domains that are common between the 2) and learn how to refine the PCC instrument and data-collection processes. Generally speaking, systematically collecting data on PCC at the national level will generate needed information on how PCC varies within and between facilities and will enable us to identify strategies for quality improvement at both the inner- and outer-hospital levels.

## Supplementary Material

qxad017_Supplementary_Data
