# Supplementary material for: Patient-centered inpatient psychiatry is associated with outcomes, ownership, and national quality measures
Source: Health Aff Sch. 2023 Jun 20;1(1):qxad017. doi: 10.1093/haschl/qxad017 (PMC10986256; doi:10.1093/haschl/qxad017)
Supplement: qxad017_Supplementary_Data [file qxad017_Supplementary_Data.zip › Supplemental Material_06112023.docx]

**Supplemental Material**

**Figure S1: Distribution of responses on the CAPE measure of patient-centered care (n = 739)**


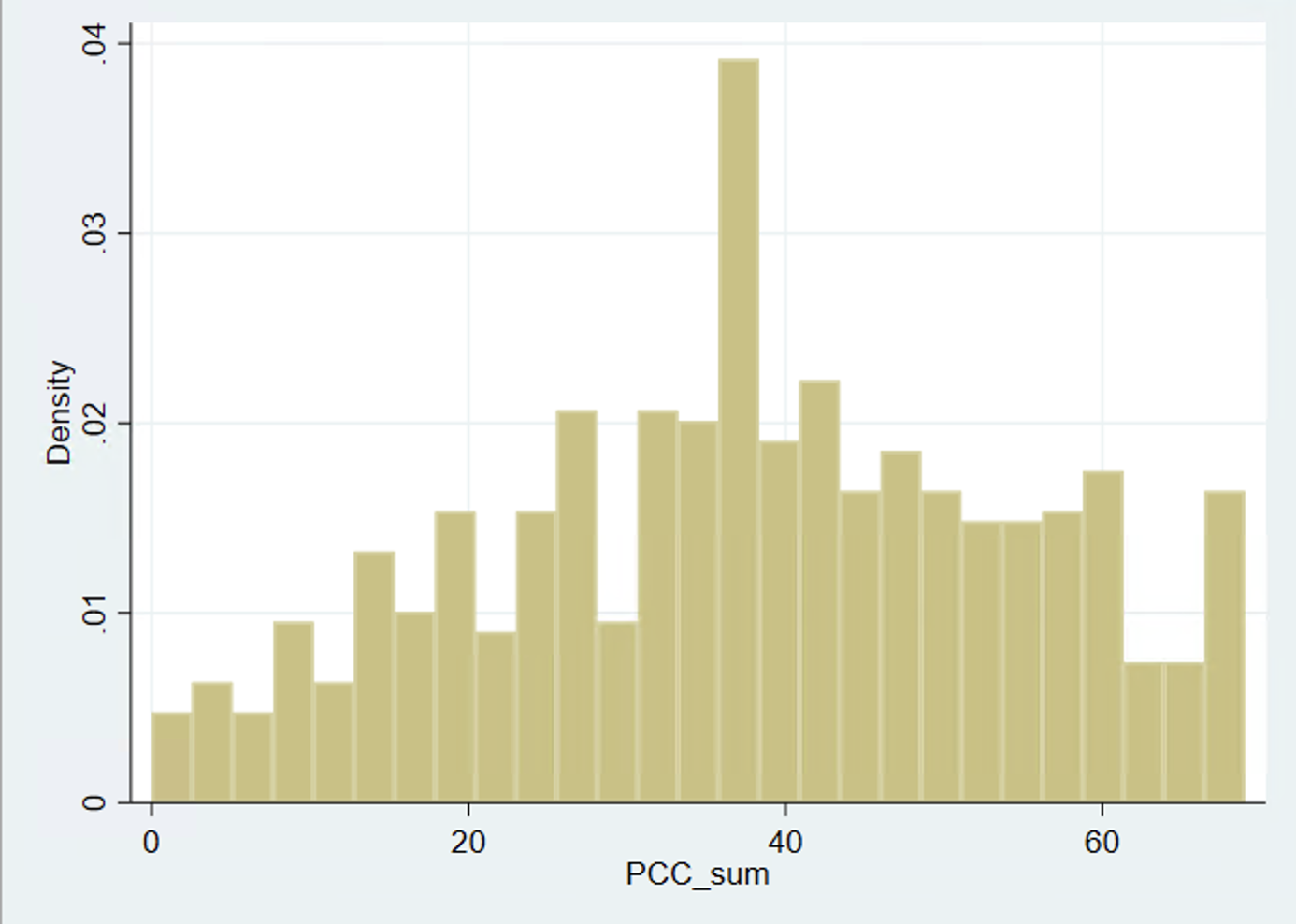


Source/Notes: SOURCE Data come from an online survey administered to former inpatient psychiatry patients in 2021.

| **Table S2: Predicting negative outcomes, full models (n = 739)** | | | | | | | | | | | | | | | | | |
| --- | --- | --- | --- | --- | --- | --- | --- | --- | --- | --- | --- | --- | --- | --- | --- | --- | --- |
|  | Hospitalization reduced trust in mental health providers | | | | Hospitalization reduced willingness to disclose distressing thoughts to outpatient providers | | | | Hospitalization reduced willingness to voluntarily seek inpatient psychiatric care | | | | Hospitalization had only a negative impact | | | |  |
|  | OR | 95% CI | | *p* | OR | 95% CI | | *p* | OR | 95% CI | | *p* | OR | 95% CI | | *p* |  |
| **Patient-Centered Care (ref = bottom 25%)** | | | | | | | | | | | | | | | | | |
| 25-50% | 0.197 | 0.120 | 0.323 | 0.000 | 0.316 | 0.194 | 0.513 | 0.000 | 0.009 | 0.001 | 0.114 | 0.000 | 0.270 | 0.159 | 0.459 | 0.000 |  |
| 50-75% | 0.118 | 0.072 | 0.193 | 0.000 | 0.223 | 0.140 | 0.357 | 0.000 | 0.003 | 0.000 | 0.052 | 0.000 | 0.145 | 0.083 | 0.253 | 0.000 |  |
| Top 25% | 0.149 | 0.089 | 0.251 | 0.000 | 0.107 | 0.061 | 0.187 | 0.000 | 0.000 | 0.000 | 0.012 | 0.000 | 0.023 | 0.007 | 0.075 | 0.000 |  |
| **Baseline expectations at time of hospitalization (ref = not at all)** | | | | | | | | | | | | | | | | | |
| Very little | 1.143 | 0.688 | 1.899 | 0.605 | 0.549 | 0.326 | 0.927 | 0.025 | 0.817 | 0.256 | 2.607 | 0.733 | 0.350 | 0.204 | 0.602 | 0.000 |  |
| A good deal | 2.007 | 1.167 | 3.449 | 0.012 | 0.725 | 0.418 | 1.258 | 0.253 | 0.489 | 0.142 | 1.684 | 0.257 | 0.251 | 0.137 | 0.459 | 0.000 |  |
| Very much | 3.389 | 1.669 | 6.884 | 0.001 | 0.564 | 0.264 | 1.206 | 0.139 | 0.904 | 0.172 | 4.742 | 0.905 | 0.198 | 0.078 | 0.500 | 0.001 |  |
| **Race (ref = Non-Hispanic White)** | | | | | | | | | | | | | | | | |  |
| Non-Hispanic Asian | 1.034 | 0.393 | 2.716 | 0.947 | 1.025 | 0.374 | 2.808 | 0.961 | 11.781 | 0.810 | 171.413 | 0.071 | 1.578 | 0.502 | 4.961 | 0.435 |  |
| Non-Hispanic Black | 0.513 | 0.199 | 1.323 | 0.167 | 1.029 | 0.403 | 2.631 | 0.952 | 0.402 | 0.040 | 4.089 | 0.441 | 1.263 | 0.457 | 3.486 | 0.653 |  |
| Non-Hispanic Native | 1.196 | 0.532 | 2.691 | 0.665 | 0.932 | 0.380 | 2.284 | 0.877 | 0.262 | 0.038 | 1.813 | 0.175 | 1.552 | 0.514 | 4.692 | 0.436 |  |
| Non-Hispanic Other | 1.183 | 0.197 | 7.113 | 0.854 | 2.669 | 0.376 | 18.936 | 0.326 | 0.182 | 0.003 | 11.266 | 0.418 | 2.812 | 0.322 | 24.569 | 0.350 |  |
| **Hispanic/Latinx** | 0.549 | 0.360 | 0.837 | 0.005 | 0.615 | 0.040 | 0.387 | 0.978 | 0.553 | 0.204 | 1.497 | 0.244 | 1.016 | 0.596 | 1.733 | 0.952 |  |
| **Gender (ref = male)** |  |  |  |  |  |  |  |  |  |  |  |  |  |  |  |  |  |
| Female | 0.899 | 0.633 | 1.275 | 0.549 | 1.504 | 0.031 | 1.037 | 2.181 | 2.286 | 0.954 | 5.475 | 0.064 | 1.240 | 0.799 | 1.925 | 0.338 |  |
| Nonbinary, third gender, other | 0.674 | 0.337 | 1.348 | 0.265 | 2.476 | 1.199 | 5.112 | 0.014 | 3.018 | 0.537 | 16.955 | 0.210 | 1.072 | 0.499 | 2.302 | 0.859 |  |
| **Age at time of admission** | 1.003 | 0.984 | 1.021 | 0.785 | 0.987 | 0.968 | 1.007 | 0.216 | 0.969 | 0.926 | 1.015 | 0.182 | 0.985 | 0.962 | 1.009 | 0.225 |  |
| **Income (ref = <$25,000)** | | | | | | | | | | | | | | | | |  |
| $25,000-$49,999 | 0.853 | 0.564 | 1.288 | 0.449 | 0.661 | 0.428 | 1.020 | 0.061 | 0.432 | 0.157 | 1.188 | 0.104 | 0.920 | 0.563 | 1.503 | 0.738 |  |
| $50,000-$99,999 | 1.389 | 0.874 | 2.207 | 0.165 | 0.919 | 0.564 | 1.499 | 0.736 | 0.860 | 0.289 | 2.558 | 0.786 | 1.068 | 0.608 | 1.873 | 0.820 |  |
| >$99,999 | 0.822 | 0.452 | 1.495 | 0.521 | 1.031 | 0.556 | 1.911 | 0.924 | 0.699 | 0.180 | 2.714 | 0.605 | 0.490 | 0.227 | 1.058 | 0.069 |  |
| **Education (ref = High School Degree or Less)** | | | | | | | | | | | | | | | | |  |
| Some college/associates degree/trade school | 0.649 | 0.403 | 1.045 | 0.075 | 0.726 | 0.439 | 1.199 | 0.211 | 0.430 | 0.139 | 1.326 | 0.142 | 1.175 | 0.649 | 2.127 | 0.594 |  |
| Four-Year College Degree | 0.708 | 0.422 | 1.186 | 0.189 | 0.663 | 0.382 | 1.150 | 0.143 | 0.095 | 0.018 | 0.501 | 0.005 | 0.757 | 0.387 | 1.478 | 0.414 |  |
| Advanced degree (master's, M.D., J.D., Ph.D. | 0.391 | 0.187 | 0.815 | 0.012 | 0.681 | 0.315 | 1.473 | 0.329 | 0.412 | 0.077 | 2.208 | 0.300 | 1.733 | 0.696 | 4.317 | 0.237 |  |
| **Had insurance** | 0.627 | 0.348 | 1.129 | 0.120 | 1.862 | 0.993 | 3.491 | 0.053 | 0.925 | 0.259 | 3.299 | 0.904 | 1.218 | 0.607 | 2.445 | 0.579 |  |
| **First hospitalization** | 0.908 | 0.643 | 1.282 | 0.584 | 1.731 | 1.207 | 2.481 | 0.003 | 1.220 | 0.544 | 2.735 | 0.629 | 1.290 | 0.847 | 1.964 | 0.236 |  |
| **Suicidality** | 0.723 | 0.504 | 1.037 | 0.078 | 0.836 | 0.568 | 1.232 | 0.366 | 1.670 | 0.682 | 4.086 | 0.262 | 1.572 | 0.989 | 2.497 | 0.055 |  |
| **Involuntary** | 1.147 | 0.806 | 1.632 | 0.445 | 1.935 | 1.342 | 2.790 | 0.000 | 1.056 | 0.461 | 2.419 | 0.898 | 1.616 | 1.065 | 2.452 | 0.024 |  |
| **Year** | 1.055 | 0.943 | 1.180 | 0.347 | 1.081 | 0.954 | 1.225 | 0.220 | 0.921 | 0.704 | 1.204 | 0.547 | 1.030 | 0.900 | 1.179 | 0.670 |  |
| Source/Notes: SOURCE Data come from an online survey administered to former inpatient psychiatry patients in 2021. The interaction between voluntary status and patient-centered care was not significant in any model; main effects models are therefore shown here. | | | | | | | | | | | | | | | | | |

| **Table S3: Predicting positive outcomes, full models (n = 739)** | | | | | | | | | | | | | | | | | | | | |
| --- | --- | --- | --- | --- | --- | --- | --- | --- | --- | --- | --- | --- | --- | --- | --- | --- | --- | --- | --- | --- |
|  | Had a 30-day follow-up visit | | | | Hospitalization increased trust in mental health providers | | | | Hospitalization increased willingness to disclose distressing thoughts to outpatient providers | | | | Hospitalization increased willingness to voluntarily seek inpatient psychiatric care | | | | Hospitalization had only a positive impact | | | |
|  | OR | 95% CI | | *p* | OR | 95% CI | | *p* | OR | 95% CI | | *p* | OR | 95% CI | | *p* | OR | 95% CI | | *p* |
| **Patient-Centered Care (ref = bottom 25%)** | | | | | | | | | | | | | | | | | | | | |
| 25-50% | 0.812 | 0.495 | 1.334 | 0.411 | 6.215 | 3.347 | 11.539 | 0.000 | 3.776 | 2.226 | 6.408 | 0.000 | 8.606 | 4.593 | 16.127 | 0.000 | 5.085 | 2.663 | 9.710 | 0.000 |
| 50-75% | 1.259 | 0.763 | 2.078 | 0.368 | 10.093 | 5.583 | 18.245 | 0.000 | 2.596 | 1.539 | 4.379 | 0.000 | 12.122 | 6.582 | 22.328 | 0.000 | 6.575 | 3.497 | 12.364 | 0.000 |
| Top 25% | 2.619 | 1.389 | 4.940 | 0.003 | 55.711 | 28.001 | 110.844 | 0.000 | 3.798 | 2.168 | 6.653 | 0.000 | 17.033 | 8.908 | 32.571 | 0.000 | 15.971 | 8.032 | 31.758 | 0.000 |
| **Baseline expectations at time of hospitalization (ref = not at all)** | | | | | | | | | | | | | | | | | | | | |
| Very little | 1.414 | 0.869 | 2.301 | 0.163 | 1.752 | 0.891 | 3.443 | 0.104 | 1.763 | 1.003 | 3.100 | 0.049 | 1.100 | 0.599 | 2.018 | 0.759 | 4.089 | 1.858 | 8.997 | 0.000 |
| A good deal | 3.098 | 1.781 | 5.389 | 0.000 | 3.220 | 1.620 | 6.403 | 0.001 | 0.956 | 0.527 | 1.734 | 0.881 | 1.100 | 0.593 | 2.041 | 0.763 | 4.100 | 1.881 | 8.936 | 0.000 |
| Very much | 3.622 | 1.629 | 8.055 | 0.002 | 3.396 | 1.424 | 8.101 | 0.006 | 0.384 | 0.172 | 0.856 | 0.019 | 0.584 | 0.267 | 1.276 | 0.177 | 1.088 | 0.430 | 2.752 | 0.858 |
| **Race (ref = Non-Hispanic White)** | | | | | | | | | | | | | | | | | | | | |
| Non-Hispanic Asian | 1.106 | 0.376 | 3.257 | 0.855 | 1.881 | 0.602 | 5.875 | 0.277 | 1.949 | 0.714 | 5.318 | 0.193 | 1.420 | 0.487 | 4.139 | 0.521 | 1.172 | 0.370 | 3.709 | 0.787 |
| Non-Hispanic Black | 1.887 | 0.604 | 5.895 | 0.274 | 1.566 | 0.494 | 4.965 | 0.446 | 2.041 | 0.767 | 5.435 | 0.153 | 2.655 | 0.881 | 8.002 | 0.083 | 0.802 | 0.229 | 2.812 | 0.731 |
| Non-Hispanic Native | 1.154 | 0.453 | 2.943 | 0.764 | 1.929 | 0.757 | 4.919 | 0.169 | 1.259 | 0.546 | 2.902 | 0.589 | 1.070 | 0.467 | 2.451 | 0.872 | 1.628 | 0.682 | 3.887 | 0.273 |
| Non-Hispanic Other | 0.177 | 0.070 | 0.027 | 1.155 | 1.518 | 0.128 | 17.991 | 0.741 | 1.349 | 0.140 | 13.001 | 0.795 | 3.275 | 0.465 | 23.072 | 0.234 | 4.765 | 0.620 | 36.608 | 0.133 |
| **Hispanic/Latinx** | 0.967 | 0.612 | 1.528 | 0.886 | 1.256 | 0.769 | 2.053 | 0.362 | 2.119 | 1.374 | 3.267 | 0.001 | 1.774 | 1.132 | 2.781 | 0.012 | 1.366 | 0.848 | 2.201 | 0.200 |
| **Gender (ref = male)** |  |  |  |  |  |  |  |  |  |  |  |  |  |  |  |  |  |  |  |  |
| Female | 1.174 | 0.798 | 1.726 | 0.416 | 0.574 | 0.377 | 0.874 | 0.010 | 0.524 | 0.359 | 0.764 | 0.001 | 0.843 | 0.573 | 1.242 | 0.388 | 1.163 | 0.773 | 1.748 | 0.469 |
| Nonbinary, third gender, other | 1.108 | 0.533 | 2.304 | 0.783 | 0.473 | 0.181 | 1.234 | 0.126 | 0.284 | 0.109 | 0.744 | 0.010 | 0.713 | 0.292 | 1.740 | 0.458 | 0.640 | 0.243 | 1.683 | 0.365 |
| **Age at time of admission** | 0.984 | 0.964 | 1.005 | 0.131 | 1.022 | 0.999 | 1.045 | 0.061 | 1.018 | 0.998 | 1.038 | 0.081 | 1.033 | 1.012 | 1.054 | 0.002 | 1.033 | 1.011 | 1.055 | 0.003 |
| **Income (ref = <$25,000)** | | | | | | | | | | | | | | | | | | | | |
| $25,000-$49,999 | 0.775 | 0.493 | 1.220 | 0.271 | 1.284 | 0.781 | 2.112 | 0.324 | 1.655 | 1.045 | 2.622 | 0.032 | 1.293 | 0.812 | 2.058 | 0.278 | 1.540 | 0.931 | 2.547 | 0.093 |
| $50,000-$99,999 | 0.658 | 0.394 | 1.099 | 0.110 | 1.318 | 0.752 | 2.310 | 0.334 | 1.907 | 1.135 | 3.204 | 0.015 | 0.814 | 0.479 | 1.384 | 0.447 | 1.295 | 0.736 | 2.280 | 0.370 |
| >$99,999 | 0.598 | 0.305 | 1.174 | 0.135 | 0.349 | 0.160 | 0.763 | 0.008 | 1.392 | 0.708 | 2.736 | 0.338 | 0.804 | 0.400 | 1.616 | 0.540 | 2.133 | 1.053 | 4.319 | 0.035 |
| **Education (ref = High School Degree or Less)** | | | | | | | | | | | | | | | | | | | | |
| Some college/associates degree/trade school | 1.444 | 0.879 | 2.370 | 0.147 | 0.751 | 0.431 | 1.309 | 0.313 | 0.661 | 0.403 | 1.083 | 0.100 | 0.839 | 0.501 | 1.405 | 0.504 | 0.348 | 0.200 | 0.604 | 0.000 |
| Four-Year College Degree | 1.848 | 1.058 | 3.230 | 0.031 | 1.732 | 0.952 | 3.154 | 0.072 | 0.509 | 0.295 | 0.879 | 0.015 | 1.465 | 0.848 | 2.530 | 0.171 | 0.540 | 0.304 | 0.961 | 0.036 |
| Advanced degree (master's, M.D., J.D., Ph.D. | 4.073 | 1.648 | 10.064 | 0.002 | 0.772 | 0.309 | 1.932 | 0.581 | 0.697 | 0.320 | 1.519 | 0.364 | 0.700 | 0.306 | 1.602 | 0.398 | 0.300 | 0.125 | 0.720 | 0.007 |
| **Had insurance** | 1.928 | 1.093 | 3.402 | 0.023 | 0.958 | 0.490 | 1.874 | 0.901 | 0.743 | 0.403 | 1.372 | 0.343 | 1.122 | 0.589 | 2.137 | 0.726 | 0.988 | 0.496 | 1.969 | 0.974 |
| **First hospitalization** | 0.907 | 0.621 | 1.324 | 0.612 | 1.063 | 0.701 | 1.612 | 0.775 | 0.630 | 0.428 | 0.925 | 0.019 | 1.155 | 0.786 | 1.698 | 0.463 | 0.883 | 0.586 | 1.332 | 0.553 |
| **Suicidality** | 0.866 | 0.580 | 1.293 | 0.481 | 0.969 | 0.631 | 1.489 | 0.887 | 1.664 | 1.116 | 2.481 | 0.012 | 1.555 | 1.041 | 2.321 | 0.031 | 1.190 | 0.786 | 1.801 | 0.411 |
| **Involuntary** | 0.978 | 0.667 | 1.434 | 0.908 | 1.079 | 0.703 | 1.654 | 0.729 | 0.946 | 0.647 | 1.383 | 0.773 | 1.427 | 0.961 | 2.119 | 0.078 | 0.891 | 0.588 | 1.350 | 0.587 |
| **Year** | 1.216 | 1.062 | 1.392 | 0.005 | 0.982 | 0.852 | 1.131 | 0.799 | 1.005 | 0.856 | 1.179 | 0.955 | 1.112 | 0.972 | 1.271 | 0.122 | 0.969 | 0.852 | 1.101 | 0.624 |
| Source/Notes: SOURCE Data come from an online survey administered to former inpatient psychiatry patients in 2021. The interaction between voluntary status and patient-centered care was not significant in any model; main effects models are therefore shown here. | | | | | | | | | | | | | | | | | | | | |

**Table S4: Mean Predicted Probabilities from fully adjusted regression models with negative outcomes (n = 739)**

|  | Hospitalization reduced trust | | | Hospitalization decreased willingness to disclose distressing thoughts | | | Hospitalization decreased willingness to go to the hospital voluntarily | | | Hospitalization had an overall negative impact | | |
| --- | --- | --- | --- | --- | --- | --- | --- | --- | --- | --- | --- | --- |
| PCC Quartiles | PP | 95% CI | | PP | 95% CI | | PP | 95% CI | | PP | 95% CI | |
| Bottom 25% | 0.76 | 0.75 | 0.77 | 0.68 | 0.66 | 0.70 | 0.92 | 0.90 | 0.94 | 0.58 | 0.56 | 0.60 |
| 26-50% | 0.44 | 0.42 | 0.46 | 0.34 | 0.31 | 0.36 | 0.37 | 0.31 | 0.42 | 0.22 | 0.20 | 0.25 |
| 51-75% | 0.35 | 0.33 | 0.37 | 0.28 | 0.27 | 0.30 | 0.23 | 0.19 | 0.27 | 0.13 | 0.11 | 0.14 |
| 76-100% | 0.46 | 0.44 | 0.48 | 0.16 | 0.14 | 0.17 | 0.04 | 0.02 | 0.06 | 0.02 | 0.02 | 0.02 |

SOURCE Data come from an online survey administered to former inpatient psychiatry patients in 2021. Predicted probabilities come from four regression models (n = 739).

**Table S5: Mean Predicted Probabilities from fully adjusted regression models with positive outcomes (n = 739)**

|  | Had a 30-day follow-up visit | | | Hospitalization increased trust | | | Hospitalization increased willingness to disclose distressing thoughts | | | Hospitalization increased willingness to go to the hospital voluntarily | | | Hospitalization had an overall positive impact | | |
| --- | --- | --- | --- | --- | --- | --- | --- | --- | --- | --- | --- | --- | --- | --- | --- |
| PCC Quartiles | PP | 95% CI | | PP | 95% CI | | PP | 95% CI | | PP | 95% CI | | PP | 95% CI | |
| Bottom 25% | 0.69 | 0.67 | 0.71 | 0.07 | 0.07 | 0.08 | 0.15 | 0.14 | 0.17 | 0.07 | 0.06 | 0.07 | 0.07 | 0.06 | 0.07 |
| 26-50% | 0.63 | 0.61 | 0.66 | 0.40 | 0.37 | 0.43 | 0.46 | 0.43 | 0.49 | 0.40 | 0.37 | 0.42 | 0.33 | 0.31 | 0.36 |
| 51-75% | 0.77 | 0.75 | 0.79 | 0.51 | 0.49 | 0.54 | 0.34 | 0.32 | 0.37 | 0.46 | 0.44 | 0.48 | 0.39 | 0.36 | 0.41 |
| 76-100% | 0.89 | 0.88 | 0.90 | 0.85 | 0.83 | 0.86 | 0.35 | 0.33 | 0.38 | 0.49 | 0.46 | 0.51 | 0.55 | 0.52 | 0.57 |

SOURCE Data come from an online survey administered to former inpatient psychiatry patients in 2021. Predicted probabilities come from five regression models (n = 739).
